# Case of Hæmorrhage from the Internal Carotid Artery Treated Successfully by the Ligature

**Published:** 1874-02

**Authors:** H. B. Sands

**Affiliations:** Surgeon to the Bellevue and Roosevelt Hospitals, etc.


*A Case of Hæmorrhage from the Internal Carotid Artery treated successfully by the Ligature.* By H. B. SANDS, M.D., Surgeon to the Bellevue and Roosevelt Hospitals, etc.

The following case is believed to be unique, and to constitute an important contribution to the annals of operative surgery.

On October 22, 1872, I performed disarticulation of the left half of the lower jaw, on account of a malignant scirrhous tumor, occurring in a gentleman, fifty-three years of age, and developed chiefly on the inner aspect of the ramus and body of the bone, near the angle. The usual incision having been made, the jaw was divided on the left of the median line, through the socket of the corresponding canine tooth. The bone was then forcibly everted, while I quickly severed, by means of a pair of stout scissors, the soft parts covering the internal surface of the tumor, and then completed the disarticulation. Copious arterial hæmorrhage, checked temporarily by the pressure of the left forefinger, attended this manœuvre, and, on subsequent examination of the tumor, there were found running through it an inch of the trunk of the external carotid artery, and portions of about equal length of the digastric muscle and the hypoglossal nerve. These parts were necessarily divided and removed, together with the tumor. The external carotid, together with some smaller arterial vessels, having been tied, I was about to close the wound, when free venous bleeding took place from a small opening that I had accidentally made in the internal jugular vein. After some reflection as to the best course to pursue, I seized the margins of the wound in the vein, and applied a lateral ligature, not occluding the calibre of the vessel. The wound was then closed by sutures, except at its middle part, where an opening, half an inch long, was left for the exit of the ligatures. During the operation it was noticed that the upper part of the common carotid, and the internal carotid artery, from its origin to the base of the skull, were exposed, and could be seen pulsating at the bottom of the wound.

On the tenth day after the operation, at ten o'clock in the evening, while my partner, Dr. Curtis, was engaged in cleaning the wound, a sudden escape of blood took place, both from the external opening and through the mouth. Dr. Curtis at once compressed the common carotid with the left hand, and ripping open the upper end of the original incision, passed in two fingers of the right hand, and made pressure over the line of the internal carotid. The hæmorrhage ceased at once, about two ounces of blood having been lost, whose color gave no certain indication of the source of the bleeding. Pressure was successfully maintained until my arrival, at the end of about half an hour. It was then found that one of the upper two fingers covered the bleeding point, which was evidently above the carotid bifurcation, and Dr. Curtis was relieved by my pupil, Mr. Shafter. The ligatures were then examined, and that on the internal jugular vein identified and found to be attached far below the bleeding point. No ligature could be identified as belonging to the external carotid artery. After looking at the ligatures, I, without giving ether, prolonged the opening in the neck downward along the anterior edge of the sterno-mastoid muscle, and endeavored to reach the common carotid high up. Owing to the altered condition of the parts, this proved impracticable; so, having divided the omohyoid muscle, I exposed the artery just below it, where the tissues were normal, and passed, without tightening it, a ligature.

The common carotid was then compressed between the ligature and the finger, and pressure relaxed upon the bleeding point. A very vigorous spurt of blood followed, and pressure was resumed.

An examination of the surface, immediately above the seat of hæmorrhage, revealed a very soft pulsation, just beneath the granulations along the line of the internal carotid. The latter vessel I directed Dr. Curtis to dissect, while I controlled the bleeding. The internal carotid was exposed by scratching through the condensed tissues with the point of a grooved steel director; a ligature was passed and was immediately tightened, as was just afterward the one encircling the common carotid. I then lifted my finger from the bleeding point, and no gush followed, but a bleeding continuous in character, and small in amount. This was easily controlled by pressure just below the opening, and for the first time the exact seat and nature of the latter were completely open to inspection. The blood was found to come from a small, circular, clean-cut ulceration in the side of the internal carotid artery, situated an inch below the upper ligature, and the same distance above the upper border of the thyroid cartilage. Through this opening, the white and glistening surface of the inner coat of the opposite side of the arterial wall was distinctly visible. After ascertaining the opening to be in the side of the internal carotid, I readily exposed this vessel two or three lines below the opening, and applied a ligature, thus cutting off the source of the trifling hæmorrhage which had persisted after the tightening of the first two ligatures. This hæmorrhage must have been caused by the recurrent circulation through branches springing from the stump of the external carotid. The lower portion of the wound was then closed by a few silk sutures, and the rest lightly filled with dry lint.

The operation, which lasted about two hours, was wonderfully well borne, the patient making no complaint. He lost altogether, both during the operation and the antecedent hæmorrhage, not more than four or five ounces of blood, and the pulse continued firm throughout. Milk and iced brandy were administered through the night, and the patient obtained sleep without anodynes.

The subsequent progress of the case was eminently satisfactory. The two ligatures on the internal carotid separated on the ninth day, that of the common carotid on the fourteenth day, and that of the internal jugular vein on the seventeenth day after their application. The upper ligature on the internal carotid had in its noose an offensive white slough of the artery, three-eighths of an inch long, and another hæmorrhage was feared. None occurred, however, and the patient recovered completely without any further unpleasant symptoms.

*Remarks.*—Lesions of the internal carotid are usually so rapidly fatal, that no opportunity is afforded for surgical treatment. But, even when the surgeon interferes, success is not generally attainable, and, so far as I have been able to ascertain, there is only one other example of recovery recorded besides the one herewith reported. This case occurred in 1807, in the practice of Dr. Twitchell, of Keene, N. H., and, in many respects, it resembled my own. The hæmorrhage was secondary, and took place, ten days after a gunshot injury, while Dr. Twitchell was in the patient's house. He applied a ligature on the cardiac side of the opening in the wall of the internal carotid, but was obliged to check the recurrent hæmorrhage by means of a graduated compress, as the opening was in that part of the artery which lies just beneath the base of the skull.

From various sources I have collected the following instances of hæmorrhage from the internal carotid. Some were treated, and others were not, while all terminated fatally:

1. A hunter received a penetrating bullet-wound of the face. Hæmorrhage occurred on the third day, after the administration of an emetic. Death took place on the fourth day, during an attempt to tie the common carotid. At the autopsy, the ball was found lying behind this vessel, opposite the bifurcation. The internal carotid showed a longitudinal rent one-fourth of an inch in length.

2. Abernethy tied the common carotid for hæmorrhage from a wound of the neck inflicted by a cow's horn. The patient died thirty hours after the operation, with symptoms of hemiplegia. At the *post-mortem* operation, the facial, lingual, superior thyroid, and internal carotid arteries were found torn.

3. Langenbeck tied the common carotid for hæmorrhage from the internal carotid, caused by the ulceration of an epithelian cancer. Death occurred soon after the operation, and an ulcer, not larger than the head of a pin, was found in the coats of the internal carotid.

4. A. Smith ligated the common carotid for hæmorrhage from the internal carotid, caused by a phagedenic ulcer of the tonsil. The patient died in six hours.

5. In the "Medical and Surgical History of the War of the Rebellion," a case is reported in which the common carotid was tied for hæmorrhage from the internal carotid, caused by a gun-shot-wound. The hæmorrhage recurred and carried off the patient.

6. Baizeau tied the common carotid for hæmorrhage from the internal carotid, caused by disease of the ear. The bleeding was not arrested, and proved fatal on the third day. At the autopsy an opening was found in the internal carotid, produced by caries of the walls of the tympanum.

7. Broca performed an operation, like the one last described, and with a fatal result due to hæmorrhage.

8. Billroth, in a case of hæmorrhage from the right ear, due to ulceration of the internal carotid, tied the right common carotid, and, a fortnight subsequently, the left common carotid. Death from hæmorrhage occurred two days after the last operation.

9. Dupuytren reports the case of a man who received a perforating bullet-wound of the neck, at the level of the inferior maxilla. Hæmorrhage, which pressure failed to arrest, occurred on the tenth day, and proved fatal on the twelfth day. At the autopsy a wound, one-half an inch in length, was discovered in the internal carotid, two inches above its point of origin.

10. Heyfelder relates that a soldier received a penetrating wound of the left side of the neck, and died of hæmorrhage eight hours after the injury. Ice-bags were the only means employed to check the bleeding. The internal carotid was found to be almost completely divided, three and a half lines above its origin.

11. Beclard states that a traveling charlatan wounded the internal carotid while attempting to excise an enlarged tonsil. The operator fled, and Beclard was summoned just in time to see the patient die from hæmorrhage. A wound of the internal carotid was found *post-mortem.*

In some of the cases above mentioned, namely, those in which the hæmorrhage was due to disease of the petrous bone, the application of a ligature on the distal side of the arterial lesion was impossible, and the case that I have reported is the only one, so far as I am aware, in which a lesion of the internal carotid has been treated by the application of a double ligature to the injured vessel, one on the proximal, and the other on the distal side of the bleeding point. The result affords additional evidence of the soundness of the rule laid down by Mr. Guthrie—a rule which is too often neglected, as is shown by the surgical reports of the late civil war, even at the present day. It is not, perhaps, difficult to explain why a surgical maxim, so generally admitted to be binding, should be so often disregarded. The application of a double ligature to the bleeding vessel is simple in principle, but generally difficult and sometimes impossible in practice. The deep situation of the bleeding vessel, its relation to other important parts, and, in cases of secondary hæmorrhage, the infiltration of the surrounding textures with inflammatory products, offer serious, and sometimes insuperable obstacles to the application of a double ligature near the opening in the arterial walls. In these circumstances, the temptation to apply a simple ligature to the main trunk is very great, and experience shows that this operation, either alone, or, as in Dr. Twitchell's case, in conjunction with pressure, may sometimes insure the desired result. Yet success in such an operation can never be expected, and the surgeon should in no case perform it except as a last resort, and after an attempt has been fairly made to apply a double ligature according to the rule admitted by nearly every surgical writer as imperative. In the present case it is plainly evident that, unless the ligature had been applied above as well as below the bleeding point, death from hæmorrhage would have rapidly and inevitably followed, as it was noticed that the simple interruption of the circulation through the common carotid produced no appreciable diminution in the violence of the bleeding, which, however, ceased almost entirely when a ligature was applied to the internal carotid beneath the base of the skull. The slight recurrent hæmorrhage still going on was controlled by the third ligature, placed just below the bleeding point. This ligature I should have applied at first, instead of tying the primitive carotid, had the state of the parts rendered the requisite dissection practicable.

Finally, it may be interesting to note the success which attended the application of a lateral ligature to the internal jugular vein. In spite of the weight of authority in favor of treating wounds of large veins by the use of a double ligature, completely surrounding the vein above and below the bleeding point, I am strongly inclined, if the wound be small, to trust to a single ligature, applied laterally, so as to include merely the edges of the wound, and not to interrupt the current of blood through the injured vessel. In case the wound were of large size, however, I should then regard the complete ligature of the vein as affording the best guarantee of success.—*New York Med. Journal.*

